# Long-term mental wellbeing and functioning after surgery for cauda equina syndrome

**DOI:** 10.1371/journal.pone.0255530

**Published:** 2021-08-06

**Authors:** James E. Hazelwood, Ingrid Hoeritzauer, Alan Carson, Jon Stone, Andreas K. Demetriades

**Affiliations:** 1 Edinburgh Spinal Surgery Outcomes Study Group, Edinburgh, United Kingdom; 2 Chelsea and Westminster Hospital, London, United Kingdom; 3 Department of Clinical Neurosciences (Neurology), Royal Infirmary of Edinburgh, Little France, Edinburgh, Scotland, United Kingdom; 4 Centre for Clinical Brain Sciences, University of Edinburgh, Edinburgh, United Kingdom; 5 Department of Rehabilitation Medicine, NHS Lothian, Edinburgh, United Kingdom; 6 Department of Clinical Neurosciences (Neurosurgery), Royal Infirmary of Edinburgh, Little France, Edinburgh, Scotland, United Kingdom; I.R.C.C.S. San Raffaele Scientific Institute, Vita-Salute San Raffaele University, ITALY

## Abstract

**Introduction:**

Cauda Equina Syndrome (CES) can cause persisting life-changing dysfunction. There is scarce literature regarding the long-term assessment of CES symptoms, and rarer still is the impact of these symptoms on mental wellbeing investigated. This study assessed the long-term patient reported mental wellbeing outcomes of post-operative CES patients.

**Methods:**

Patients who underwent surgery for CES between August 2013 and November 2014 were identified using an ethically approved database. They then completed validated questionnaires over the telephone assessing their mental and physical functioning (Short-Form 12 Questionnaire), generating the Physical Component Summary (PCS) and Mental Component Summary (MCS). Bladder, bowel and sexual function were also assessed using validated questionnaires. MCS scores were compared to both the Scottish mean and previously published cut-offs indicating patients at risk of depression. Correlations of MCS with bladder, bowel, sexual and physical dysfunction were examined and multifactorial regression to predict MCS from these variables analysed. Independent t-tests assessed the mean difference in MCS between patients presenting with incomplete CES (CES-I) and CES with retention (CES-R) and between those with radiologically confirmed and impending CES.

**Results:**

Forty-six participants with a mean follow-up time of 43 months completed the study. The mean (±SD) MCS was 49 (±11.8) with 22% demonstrating poor mental health related quality of life in comparison to the Scottish mean. Overall, 37% had scores consistent with being at risk for depression with in the last 30 days, and 45% within the last 12 months. MCS was significantly correlated with Urinary Symptoms Profile (USP) score (-0.608), NBDS score (-0.556), ASEX score (-0.349) and PCS score (0.413) with worse bladder, bowel, sexual and physical dysfunction associated with worse MCS score. Multifactorial regression analysis demonstrated both urinary (USP score p = 0.031) and bowel function (NBDS score p = 0.009) to be significant predictive variables of mental health related quality of life. There were no significant mean differences in MCS between those presenting with CES-I and CES-R or those with radiologically complete and impending CES.

**Discussion:**

This study demonstrates a high frequency of being at risk for depression in patients with CES and identifies outcome measures (physical, sexual and more so bladder and bowel dysfunction) associated with poorer mental wellbeing. Our large cohort and long follow-up highlight that CES patients should be considered at risk of depression, and the need to consider mental health outcomes following CES surgery.

## Introduction

Cauda Equina Syndrome (CES) can cause persisting, life-changing dysfunction across multiple body systems. Due to the range of structures innervated by the nerves of the cauda equina, compressive pathology in this area causes transient or permanent bladder, bowel and sexual dysfunction, and sensory, motor and reflex dysfunction of the perineum and lower limbs [1].

The incidence of CES is difficult to accurately determine, largely due to inconsistency in the evidence base regarding its definition, and use of ambiguous subclassifications [1]. A recent systematic literature review found an incidence of 0.3–0.5/100,000 in asymptomatic community populations and in 0.27% of those presenting to secondary care with lower back pain, although authors remark that comparisons between studies are hindered by heterogenous definitions [2]. One such definition is “impending CES”, whereby a patient complains of radicular symptoms and saddle numbness, in the absence of sphincter dysfunction but with a confirmatory radiological evidence of cauda equina compression.

However, there is scarce literature regarding the long-term assessment of outcomes following CES surgery, especially that which *comprehensively* assesses the multi-faceted symptomatology that can be present. Rarer still is the investigation of the impact of these wide-ranging symptoms on mental wellbeing in this population. In their systematic review of outcomes reported in analyses of patients following CES, Srikandarajah *et al*. demonstrate that although bladder or physical function are commonly assessed (in 70.5% and 63.9% of papers respectively), sexual function is less so (26.2%), probably due to reluctance of health professionals to enquire despite its significant burden [3–5]. Even when these variables are assessed, often it is as a dichotomic function/dysfunction assessment, as opposed to assessing severity of dysfunction as a patient reported outcome (PROM) [6]. Further still, emotional functioning is rarely investigated–only assessed in 6.6% of papers studying outcomes after CES, with only 4.9% stating the assessment tool used and dysfunction defined in only 1.7% [3]. This is disheartening in the context of a recent international patient and healthcare professional consensus meeting, which concluded that social functioning, low mood and depression are outcomes of maximal importance to this population [7].

We have previously reported bladder, bowel, sexual and physical function outcomes using validated questionnaires in a cohort of post-operative CES patients [6]. In this current study we examine mental health outcomes in this same cohort, which also represents a selected subgroup of the patients included in a previous study by Hoeritzauer *et al*. [8].

An additional assessment of *mental health related quality of life* after surgery in this population has the potential to provide great value to this patient group, as would analysis of factors correlating with reduced mental functioning, which to our knowledge have not yet been explored.

### Aims

Using validated questionnaires, this study assessed the long-term post-operative mental *health related quality of life* of patients following surgery for CES. A secondary aim was to assess bladder, bowel, sexual and physical function and to correlate the effects of dysfunction in these domains on mental functioning.

## Materials and methods

We used our institutional database for patients with cauda equina syndrome, which has previously been described in both Hoeritzauer *et al*. and Hazelwood *et al*. [6, 8]. The data regarding psychiatric co-morbidity was previously reported in the Hoeritzauer *et al*. dataset, of which this patient group is a selected subgroup [8]. The novel aim in this present study was to analyse specifically the mental health outcomes following diagnosis of cauda equina syndrome, and predictive factors thereof. The current study complied with the Declaration of Helsinki and was approved by the Institutional Review Board of Edinburgh University (SSC5a/1305797). Informed consent was obtained verbally, but signed forms were waived due to the retrospective nature of the study and anonymized database.

77 patients who had undergone decompressive surgery for CES caused by degenerative and/or prolapsed intervertebral disc disease between August 2013 and November 2014 were identified from a tertiary neurosurgical centre departmental database. All procedures were performed by the same department and consisted of microdiscectomy via laminectomy or laminotomy. Participants were excluded from the study in cases of CES caused by pathology other than degenerative disc disease. They were also excluded if they were unable to complete the questionnaire through insufficient English, untraceable contact details, or death. Following identification, participants were contacted by telephone and completed the questionnaire, providing point-prevalence data for long-term outcomes in these patients. Electronic medical records were used to supplement information from the questionnaire regarding age, gender and any co-morbid psychiatric diagnoses documented up to the time of follow-up. All patients had been offered standard of care and referral to other specialties if required, with physiotherapy, urology and pain medicine the most common, as previously reported [6]. Informed consent was obtained from all participants included in the study prior to commencement of questionnaire with ethical approval for the study granted by the University of Edinburgh.

### Measures

Post-operative Mental and Physical functioning were assessed using the Short-Form 12 (SF-12) Mental Component Summary (MCS) and Physical Component Summary (PCS) respectively, a widely validated measure of health-related functioning. Post-operative bladder dysfunction was defined by the Urinary Symptoms Profile score ≥1, Bowel dysfunction by the Neurogenic Bowel Dysfunction Score ≥6 and Sexual dysfunction by the Arizona Sexual Experiences Scale with overall score ≥19, one domain >5, or 3 domains ≥4 [9–11].

### Statistical analysis

Cohort MCS scores were compared to Scottish Adult mean values to determine the range of dysfunction present [12]. Mental health dysfunction was defined a) by an MCS score less than the Scottish mean minus one standard deviation and b) using the cut-offs described in a study of over 21000 individuals [13]. This study concluded that an MCS score of under 45.6 represents participants with a high sensitivity (SN) and specificity (SP) for depression criteria using diagnostic interview within the previous 30 days (AUC = 0.92, SN 0.86, SP 0.88) and under 48.9 for depression within previous 12 months (AUC = 0.85, SN = 0.87, SP = 0.86).

Descriptive statistical analysis of these variables was performed, and the Pearson Product Moment Correlation Coefficient was used to assess the correlation of MCS score with bladder, bowel, sexual and physical dysfunction, and with time to patient follow-up.

A multifactorial regression was then run to predict MCS score from bladder, bowel, sexual and physical dysfunction scores.

Independent T-tests were used to assess the mean differences in MCS score between males and females and those presenting with radiologically confirmed or impending CES, whereby a patient complains of radicular symptoms and saddle numbness, in the absence of sphincter dysfunction but with a confirmatory radiological evidence of cauda equina compression. Similarly mean difference in MCS score was assessed between those presenting with incomplete CES (CES-I) with saddle anaesthesia and loss of desire to void, and CES with retention (CES-R) with painless urinary retention and overflow incontinence [14]. A subgroup analysis of the impending and complete CES populations was performed using t-tests to compare the outcomes in all variables between these two groups. Lastly, a one-way analysis of variance (ANOVA) was used to assess mean differences in MCS score between those who underwent decompression <24 hours, 24–48 hours or >48 hours from symptom onset.

Statistical Analysis was performed using SPSS version 24 for Mac OS X (SPSS Inc., Chicago, IL) with statistical significance determined by *p*-value <0.05.

## Results

46 of 77 eligible participants completed the study protocol, 19 males and 27 females (inclusion rate 60%). The mean age of the group was 45.4 (Range 21–83, SD ± 14.1) years and mean time from admission to follow-up was 43.4 months (Range 36–50, SD ± 4.43).

Overall, 23.9% (n = 11) were documented as having at least one psychiatric co-morbidity at follow-up, with 15.2% (n = 7) having a diagnosis of anxiety and 21.7% (n = 10) having a diagnosis of depression.

### Emotional functioning

**Fig 1:**
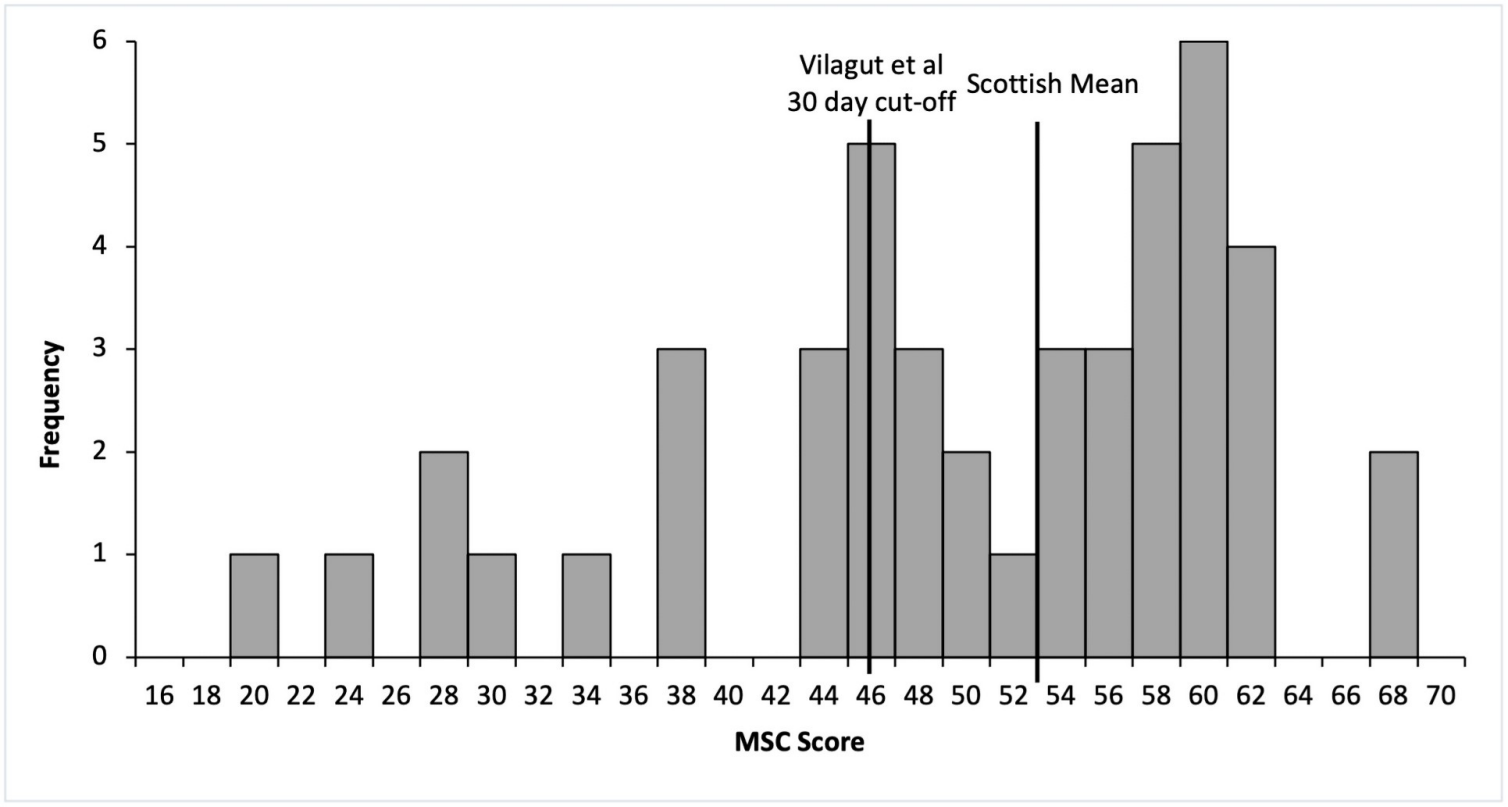
Histogram of patient SF-12 MCS distribution. Scottish adult mean MCS score = 52, Vilagut *et al*. MCS cut-off for risk of depression within 30 days = 45.6.

The SF-12 MCS demonstrated 22% (n = 10) of patients to have a statistically significant reduction in mental health related quality of life in comparison to the Scottish adult mean (±SD) of 52 (±8.8). The mean (±SD) MCS score in this cohort was 49.0 (±11.8) and median score 51.5. The upper quartile was 58.2 and lower quartile 43.7 with an interquartile range of 14.5 and range of 47.7 (Fig 1). Using the Vilagut *et al*. MCS cut-offs, 37% (n = 17) of patients had MCS values which put those patients at risk of depression within the past 30 days, and 45% (n = 20) had MCS values associated with risk of depression within the past 12 months.

Of the patients with a significant reduction in MCS score, only 2/10 had been identified by the healthcare system as having co-morbid psychiatric diagnosis by the time of follow-up.

### Dysfunction of other systems

As previously reported, bladder dysfunction was present in 76.1% (n = 35) of participants as defined by the USP. Bowel dysfunction was present in 13% (n = 6) of participants as defined by the NBDS. Sexual dysfunction was present in 39.1% (n = 18) of participants as defined by ASEX, and physical dysfunction in 47.8% (n = 22) in comparison to Scottish adult mean [6]. As reported previously, bladder, bowel and sexual outcomes were worse in those with CES-R than CES-I and bowel dysfunction was the only variable worse in true than impending CES (p = 0.027).

### Influencing factors

There was no significant relationship demonstrated between MCS score and age (r = 0.164, p = 0.277), or time to follow-up (*r* = -0.161, p = 0.295) (Table 1).

**Table 1:** Pearson-product moment correlation coefficient between MCS and other variables.

Variables	*r* =	*p* = (* = <0.05)	Relationship
PCS	0.413	0.004*	Moderate positive
USP	-0.608	0.000*	Moderate negative
NBDS	-0.556	0.000*	Moderate negative
ASEX	-0.349	0.017*	Moderate negative
Age	0.164	0.277	Nil
Time to follow-up	-0.161	0.295	Nil

Note–MCS = Mental Component Summary, PCS = Physical Component Summary, USP = Urinary Symptoms Profile, NBDS = Neurogenic Bowel Dysfunction Score, ASEX = Arizona Sexual Experiences Scale.

There was statistically significant, moderate strength, negative correlation between MCS and USP score (r = -0.608). Worse bladder dysfunction correlated with worsening MCS score. This was specifically in the domains of overactive bladder function (r = -0.569) and stress incontinence (r = -0.617), and not the poor stream domain (r = -0.149, p = 0.325).

There was also a statistically significant, moderate strength, negative correlation between MCS and NBD score (r = -0.556), with MCS worsening as bowel dysfunction worsened.

Similarly, there was a statistically significant, moderate strength, negative correlation between MCS and ASEX score (r = -0.349), with worsening sexual dysfunction associated with worse MCS.

There was a statistically significant, moderate strength, positive correlation between MCS and physical functioning, with MCS improving as physical function improves (r = 0.413, p = 0.004).

### Regression

A significant regression equation to predict MCS score from the above measured variables ((F4,41) = 9.707, p < .000) with an R^2^ of 0.486 was calculated and explains 48.6% of variance in MCS. Participants predicted MCS is equal to 47.120–0.562 (USP)– 0.967 (NBDS). MCS decreased 0.562 points for each point of USP and decreased 0.967 points for each point of NBDS. Increasing USP score and NBDS were significantly associated with a lower MCS Score.

### Analysis by groups

There was no significant mean difference in MCS score between male and female participants, or in patients undergoing decompression <24 hours, 24–48 hours or >48 hours after symptom onset. There was also no significant mean difference in MCS score between patients presenting with true and impending CES, or those presenting with CES-I and CES-R.

There were 16 patients presenting with incomplete CES and 30 presenting with true CES.

There was no significant mean difference in long term outcomes for bladder, sexual or physical function between those with impending and true CES, but there were significantly poorer outcomes present in bowel function for those with true CES (p<0.001).

## Discussion

This study aimed at assessing the long-term mental functioning in patients following surgery for CES; and at investigating additional outcome measures associated with poorer mental functioning in a population of previous CES patients, using validated questionnaires. Results demonstrate the range of mental health related quality of life in this population and the high prevalence of those with significantly low scores when compared to population mean values and cut offs, signifying a population at risk of depression. Of these patients with low scores, very few had been identified as having a current psychiatric diagnosis throughout follow-up. There were significant associations between worsening bladder, bowel, sexual and physical dysfunction and poorer mental health related quality of life. This trend is further supported by regression modelling demonstrating that mental health related quality of life could be significantly predicted using urinary and bowel dysfunction scoring. This suggests that of all domains measured, these are the most significant to mental health related quality of life and so could have role in identifying those in need of mental health support.

Few studies have directly assessed the mental functioning of patients following CES surgery. When this has been done, often authors have used the SF-36 questionnaire, from which the SF-12 was derived, allowing a degree of comparative analysis. Busse *et al*. assessed quality of life using the SF-36 at 4 months in 10 patients who underwent decompression for CES. They found decreased scores across all domains, significant in pain, social function and role physical, though not statistically significant in emotional role or mental health, possibly due to small sample size, as 7 patients mental health and 5 emotional function were below normal limits [15]. In a larger study of 42 patients, McCarthy *et al*. assessed quality of life using the SF-36, finding the aggregate scores of the population of post-operative CES patients to be outside of normal limits regarding social and physical function, physical and mental role, pain and general health, but not for mental or energy domains [16]. These broadly agree with our findings in that such a patient population as a whole does not have significantly reduced mental health related quality of life when the mean is analysed, using the MCS as a proxy PROM for mental health related quality of life. However, McCarthy *et al*. do not report the SF-36 scores on an individual basis and so does not quantify the frequency of individual patients scoring outside of normal limits, and so comparisons are unable to be made in this aspect.

The distribution of long-term mental health outcomes in post-operative CES is not well described thus far, and to our knowledge, no work has examined bladder, bowel, sexual and physical function and the relationship of these variables to mental health related quality of life in this population. Subgroup analysis demonstrated similarity in long-term functional outcome between impending and true CES in all domains except for bowel dysfunction, where true CES patients had poorer outcomes. However, we would argue that when analysing long-term outcomes in the post-CES patients, impending should be included alongside true CES patients, given its position as an important entity and the working environment of the UK healthcare system whereby investigation and management of such cases is required. In our study worse dysfunction across all variables measured was associated with a decreased MCS score. Interestingly, although both overactive bladder, and stress incontinence were significantly related, poor stream was not. Anecdotally, patients scoring highly in poor stream were using self-catheterisation to prevent overflow incontinence and as such had a measure of control over their urinary output, perhaps improving their MCS score. Given the importance of bladder and bowel demonstrated by the regression equation, future work could analyse in detail the subset of patients with more severe dysfunction, with the aim of clarifying whether particular strategies are associated with improved mental health outcomes. As reported above, there is a significant burden of bladder dysfunction in this post-operative population. This agrees with the findings of Sangodimath *et al*. who demonstrate with urodynamic studies that 100% of their patient group presented with abnormal detrusor function, and 47% remained abnormal at follow-up [5]. This shows objective impairment of bladder function, correlating with our own findings of patient-reported urinary dysfunction, and providing a plausible mechanism to explain the significant impact of mental health related quality of life by this variable. They also report high rates of sexual dysfunction using alternative questionnaires, which although not directly comparable with our results, demonstrate sexual dysfunction to be consistently present post-operatively in this population, and hence the impact of CES on mental health.

In their analysis of clinical features of 276 scan positive and scan negative CES patients, Hoeritzauer *et al*. found from patient records that 22% of the scan positive patients had co-morbid depression prior to, or within 16 months of developing CES [8]. However, as previously discussed this dichotomic assessment of outcomes does not fully assess the severity of mental dysfunction and represents a short follow-up period post-operatively. Furthermore, the majority of the literature focuses on the concept of poor mental health as a pre-operative variable associated with poorer outcomes, as opposed to measuring it as a patient reported outcome post-operatively in its own right. A number of studies demonstrate that pre-operative depression confers worse outcomes in spinal surgery. In a study of over 70,000 patients undergoing lumbar spine procedures, Schoell *et al*. demonstrated pre-operative depression to increase the risk of post-operative neurological complications [17]. Further, Sinikallio *et al*. found those with pre-operative depression showed poorer improvements in disability, pain and walking at 3 months follow-up for spinal stenosis surgery, agreeing with Chaichana *et al*. who in their population of 67 patients undergoing discectomy found both pre-operative depression and somatic anxiety decreased the likelihood of the operation improving patient disability or quality of life at 12 months [18, 19].

However, the frequency of co-morbid depression in a cohort of patients following surgery for CES has rarely been described; Gibson *et al*. reported this to be present in only 8% (n = 2), whereas Hoeritzauer *et al*. described a frequency of 22% (n = 17) in their study (whose population includes this present cohort) [8, 20]. However, although the frequency of documented co-morbid psychiatric disorder was low in the Gibson study, it was higher (30%) when self-reporting. Our study broadly agrees with the above, as in our cohort, 21.7% (n = 10) had documented evidence of depression–a greater prevalence than the Scottish population average of 13% [21]. Our analysis of the MCS data suggests 22% (n = 10) have reduced mental health outcomes at follow-up in comparison to the Scottish mean, with 37% reporting a score consistent with those that would fulfil depression criteria within the past 30 days, and 45% within the last year, thereby agreeing with Gibson *et al*. that the frequency of documented diagnoses is low, and higher using a self-reported method. Furthermore, only 20% (n = 2) of those identified as having a reduced mental health related quality of life using the MCS score were documented as having a co-morbid psychiatric diagnosis at follow-up. Questionnaires do tend to overestimate depression frequency but nonetheless this may suggest a degree of under-diagnosis and under-treatment of a portion of this population with currently unmet psychosocial needs who could benefit from assessment and intervention [22, 23].

Although not representative of a post-CES population, in a study of 201 patients following a traumatic spinal cord injury Dryden *et al*. demonstrated that 28.9% of spinal cord injury patients were treated for depression within 6 years of sustaining the injury [24]. Similar to our findings, they found that patients were at a higher risk of developing depression if they had a permanent neurological deficit, which was equal in hazard rate ratio to patients having a history of substance abuse or pre-injury history of depression. The authors make a recommendation in advocating that these risk factors could be used to target patients for assessment and support within the hospital and community settings. The screening for mental health conditions around the time of spinal surgery has a precedent in further papers, albeit in reference to its effect if present pre-operatively. As discussed, both Schoell *et al*. and Sinikallio *et al*. found an association with pre-operative depression and poorer operative outcomes, and as such both also advocate for depression screening to be a component of pre-operative assessment in these population [17, 18].

Future work in this area could continue to assess this cohort at time intervals, to investigate whether MCS scores improve or decrease over time. Studies could analyse anxiety and depression in detail using verified scales such as the HADS, GAD, Zung Scale or PHQ9 questionnaires, and use medical records to assess prescription of psychotropic medication and whether this changes over time. Previous work such as Kaptan *et al*. have demonstrated a correlation between pain scores and depression in patients with spinal stenosis [25]. Given this finding, further work could also include an assessment of pain using a validated questionnaire in a post-surgical CES population, again facilitating the monitoring of this variable over time.

### Limitations

Our study is limited by its small sample size and 60% questionnaire response rate. This confers a risk of selection bias, however, given the highly personal nature of the topics discussed during the interview, we consider this inclusion rate to be satisfactory. This study is strengthened by its size, given that the median number of CES patients in prior studies is only n = 14 [3]. Additionally, to our knowledge this represents the study with the largest population with long term follow-up of CES patients.

Furthermore, the use of a semi-structured interview approach aided a limitation of social desirability bias. Patient reported outcomes provide a more nuanced assessment of dysfunction with greater real-world relevance but at higher risk of bias than objective measurement. In addition, as a point-prevalence study, we have no measure of participants’ pre-morbid functioning, so cannot be certain that all of the outcomes demonstrated are related to CES. In light of the rarity of CES rendering a prospective design challenging, the pragmatic study design was chosen, though with no feasible method to calculate pre-morbid status. As such, in lieu of this we have compared participant MCS scores to the Scottish adult mean.

## Conclusion

At 43.4 months mean follow-up, this long-term assessment of mental functioning of CES patients after surgery demonstrates the range of mental health related quality of life in this population. It describes the high frequency of those with significantly low scores in this domain and therefore high likelihood of high rates of depression in CES patients. Of this group, few had been previously identified as having a co-morbid psychiatric diagnosis, suggesting an unmet need for psychological assessment and management.

This study also identifies that mental dysfunction in this population is significantly correlated with dysfunction in the domains of bladder, bowel, sexual and physical function. Further, that bladder and bowel dysfunction scoring are significant in regression modelling suggests a trend whereby these variables can be used to predict mental health related quality of life. This provides novel data regarding long-term mental health outcomes in this condition and we hope this will provide information to better identify those at risk and perhaps allow targeted mental health support for those in need.

## Supporting information

S1 Data (XLSX)

## Abbreviations

CES: cauda equina syndrome
MCS: Mental Component Summary (MCS)
PCS: Physical Component Summary (PCS)
SF12: Short-Form 12 Questionnaire
